# The Role of Mitochondrial Permeability Transition in Bone Metabolism, Bone Healing, and Bone Diseases

**DOI:** 10.3390/biom14101318

**Published:** 2024-10-17

**Authors:** Xiting Zhu, Ziqi Qin, Min Zhou, Chen Li, Junjun Jing, Wushuang Ye, Xueqi Gan

**Affiliations:** State Key Laboratory of Oral Diseases, National Clinical Research Center for Oral Diseases, West China Hospital of Stomatology, Sichuan University, Chengdu 610041, China; zhuxiting@stu.scu.edu.cn (X.Z.);

**Keywords:** mitochondrial permeability transition pore, mitochondrial dysfunction, mitochondria, bone healing, osteoporosis, inflammatory bone loss

## Abstract

Bone is a dynamic organ with an active metabolism and high sensitivity to mitochondrial dysfunction. The mitochondrial permeability transition pore (mPTP) is a low-selectivity channel situated in the inner mitochondrial membrane (IMM), permitting the exchange of molecules of up to 1.5 kDa in and out of the IMM. Recent studies have highlighted the critical role of the mPTP in bone tissue, but there is currently a lack of reviews concerning this topic. This review discusses the structure and function of the mPTP and its impact on bone-related cells and bone-related pathological states. The mPTP activity is reduced during the osteogenic differentiation of mesenchymal stem cells (MSCs), while its desensitisation may underlie the mechanism of enhanced resistance to apoptosis in neoplastic osteoblastic cells. mPTP over-opening triggers mitochondrial swelling, regulated cell death, and inflammatory response. In particular, mPTP over-opening is involved in dexamethasone-induced osteoblast dysfunction and bisphosphonate-induced osteoclast apoptosis. In vivo, the mPTP plays a significant role in maintaining bone homeostasis, with many bone disorders linked to its excessive opening. Genetic deletion or pharmacological inhibition of the over-opening of mPTP has shown potential in enhancing bone injury recovery and alleviating bone diseases. Here, we review the findings on the relationship of the mPTP and bone at both the cellular and disease levels, highlighting novel avenues for pharmacological approaches targeting mitochondrial function to promote bone healing and manage bone-related disorders.

## 1. Introduction

Bone is a dynamic organ that undergoes lifelong metabolism, a process encompassing both bone formation and resorption [1,2]. In the physiological state, these processes are in a dynamic balance and work together to maintain bone homeostasis. Disruption of this balance often leads to the development of degenerative and inflammatory diseases of the bone [3]. Active bone metabolism is also involved in bone damage repair and bone regeneration.

As important organelles in eukaryotic cells, mitochondria have a variety of interrelated functions, producing ATP and many biosynthetic intermediates, while also participating in cellular stress responses and the organism’s physiology. Mitochondrial dysfunction has emerged as a key factor in multiple diseases, such as neurodegenerative disorders, cardiomyopathies, metabolic syndrome, cancer, and obesity [4]. Bone has high energy demands and is, therefore, expected to be highly sensitive to mitochondrial dysfunction.

As research into mitochondrial regulation of the cellular state and microenvironment progresses, the importance of the mitochondrial permeability transition pore (mPTP) has gained increasing attention. The mPTP is a low-selectivity channel assembled at the interface of the inner and outer mitochondrial membranes. Opening of the mPTP permits the abrupt flux of low-molecular-weight solutes (molecular weight up to 1.5 kDa) across the normally impermeable inner mitochondrial membrane (IMM), known as mitochondrial permeability transition (mPT) [5]. The mPTP is actively involved in a variety of cellular events, including regulated cell death (RCD) activation, differentiation commitment of stem cells, and signalling pathways. The critical role of the mPTP in the microenvironment of the heart, the brain, and cancer has been extensively investigated and validated. Recent studies have also revealed its role in bone tissue, but there is currently a lack of reviews concerning the correlation between the mPTP and bone. This review aims to delineate the involvement of the mPTP in bone metabolism, bone healing, and bone repair, with the expectation of providing a novel perspective for targeting mitochondrial function to regulate bone homeostasis.

## 2. Overview of the mPTP

Opening of the mPTP plays a crucial role in mitochondrial function and cellular signalling, triggering various responses ranging from the physiological regulation of mitophagy to the activation of apoptosis or necrosis.

Although there are multiple candidate molecules for the mPTP, its molecular nature remains debatable. The voltage-dependent anion channel (VDAC), translocator protein (TSPO), and mitochondrial inorganic phosphate carrier (PiC) have been proposed as molecular components of the mPTP, but none met the challenge of genetic deletion modelling [6,7,8]. Adenine nucleotide transporter (ANT) was long regarded as a component of the mPTP but was demoted to a regulator as mPTP activity persisted in mitochondria from *Ant1* and *Ant2* double-null mice [9]. However, recent inner-membrane patch clamping of mitochondria from *Ant1*, *Ant2*, and *Ant4* triple-null mice revealed that embryonic fibroblasts lacking all three ANT isoforms completely lack mPTP activity [10]. This suggests that ANT directly constitutes a fundamental unit of mPTP activity in certain cell types. In the past decade, the F_1_F_o_ ATP synthase has received much attention and is proposed as a pore-forming component based on several evidence chains [11]. One proposed model suggests that the mPTP forms at the interface between monomers within the ATP synthase dimer [12]. Another model posits that the c-ring in the Fo region constitutes the pore [13,14], with the F_1_ subcomplex functioning as its gate [15]. Pekson et al. [16] recently demonstrated that the loss of an assembled mitochondrial ATP synthase due to the deletion of subunits g and F6 sensitises mPTP opening, suggesting that ATP synthase acts as a negative regulator rather than a component of the mPTP. However, this perspective was subsequently challenged by Salvatore Nesci [17]. Using advanced refractive imaging techniques, Pavlov et al. reported that both the ANT and the peripheral stalk of the F_1_F_o_ ATPase are essential for the formation of a large mPTP [18], whereas the c subunit of ATP synthase may be non-essential [19].

**Figure 1:**
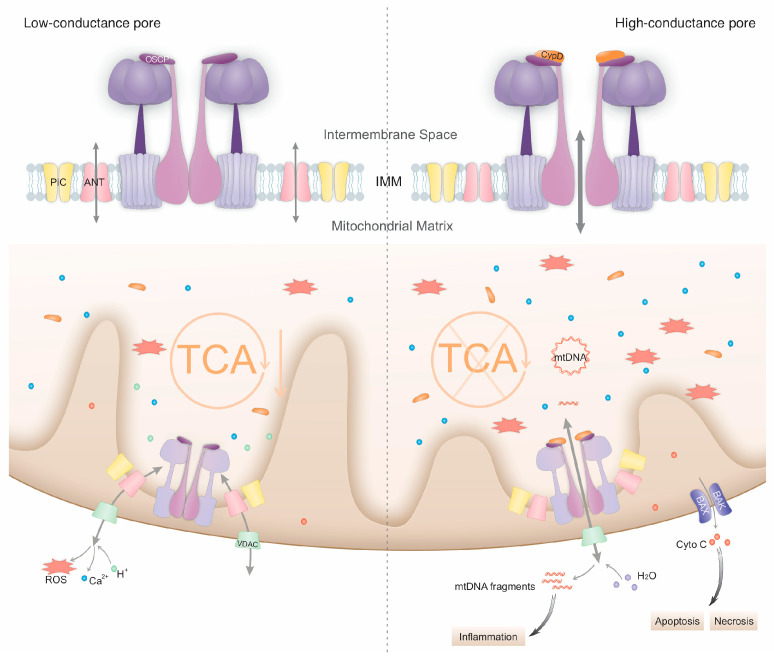
mPTP structure and the consequences of mPTP opening. The mitochondrial permeability transition pore (mPTP) has different conductance states. In its low-conductance state, the mPTP, potentially formed by the adenine nucleotide translocator (ANT), allows for the passage of ions and small metabolites. Ca^2+^ efflux in this state limits the Ca^2+^-dependent tricarboxylic acid (TCA) cycle. In its high-conductance state, the mPTP is formed by a rearrangement of the F_1_F_o_ ATP synthase complex. This state has more detrimental effects on cellular function. The release of mitochondrial DNA (mtDNA) through the mPTP triggers inflammatory responses. Extensive water influx causes mitochondrial swelling and subsequently induces outer membrane permeabilisation and the release of pro-apoptotic cofactors, leading to apoptosis or necrosis and TCA cycle collapse. ↓(orange downward arrow) represents downregulation of the TCA cycle.

Despite the absence of a consensus regarding the various potential components, the idea of a “multi-pore model” [10] has become dominant. The most plausible model so far indicates that there are two forms of the mPTP: the low-conductance pore and the high-conductance pore (Figure 1). The low-conductance pore allows for the redistribution of ions and small metabolites and is mainly involved in physiological changes in mitochondria, while the high-conductance one allows for the passage of larger solutes and has a greater effect on mitochondrial function, tending to trigger regulated cell death (RCD). Opening of the low-conductance and high-conductance mPTP might be generated by ANT and the rearrangement of the F_1_F_o_ ATP synthase, respectively [10,11,14,20,21,22].

### 2.1. mPTP Activation and Modulation

It is well established that mitochondrial Ca^2+^ is the trigger for mPTP opening [23,24,25]. Meanwhile, a variety of endogenous molecules can regulate the sensitivity of mPTP opening by modifying the threshold for the Ca^2+^ concentration required to induce mPT. Endogenous modulators involved in Ca^2+^-induced mPTP opening include inhibitors such as ADP, ATP, H^+^, NADH, and Mg^2+^ and activators such as fatty acids, inorganic phosphate, and reactive oxygen species (ROS) [11,26]. A reduction in mitochondrial membrane potential predisposes the mPTP to spontaneous opening [11].

Investigations of the pathophysiological effects of mPTP opening were mainly based on the discovery of cyclosporine A (CsA), a prototypical inhibitor of mPT [24]. Initial studies revealed that CsA promoted mitochondrial calcium accumulation due to its inhibition of the mPTP [27,28]. Subsequently, cyclophilin D (CypD) was proposed as the target of CsA during its blockade of the pore [29]. The use of CsA to inhibit CypD and, thereby, suppress mPT was demonstrated to have protective effects against cellular dysfunction caused by various stimuli, such as inflammation and oxidative stress [30,31,32]. These protective effects were validated in experimental models across multiple organs, including the heart, brain, and kidney [33,34,35]. Notably, CsA is also known to inhibit phosphatase calcineurin and other cyclophilins, which should be considered when studying CsA-induced CypD downregulation [36]. For instance, CsA reduces chemoresistance in lung cancer cells by inhibiting the Ca^2+^/calcineurin/ERK pathway [37] but enhances the tumorigenic potential of the skin through suppression of calcineurin/NFAT signalling [38]. CypA, another protein of the cyclophilin family, was found to enhance the growth of cancer stem cells via interaction with CD147 [39] and upregulation of Wnt/β-Catenin signalling [40], while CsA blocks these processes.

Encoded by the nuclear gene *Ppif*, CypD is the only genetically proven opener of the mPTP [11,41,42]. The mechanism by which CypD regulates the mPTP may involve its binding to oligomycin sensitivity-conferring protein (OSCP) subunits at the lateral stalk of the ATP synthase [12,43,44], thus reducing the threshold for mPTP opening and improving opening probability. Multiple post-translational modifications, such as deacetylation, oxidation, and S-nitrosylation, were demonstrated to regulate CypD activity, which, in turn, regulates mPTP opening [45]. The desensitisation of the mPTP by deacetylating CypD on K166 by Sirtuin 3 (SIRT3) was shown to provide a protective effect in cardiac ischaemia/reperfusion injury [46,47] and neuropathic pain [31]. Conversely, the absence of this modulatory effect was found to exacerbate age-related periodontal bone resorption in mice [48]. On cysteine 203 of human CypD or homologous cysteine 202 in mice, oxidation sensitises the mPTP, whereas S-nitrosylation, S-glutathionylation, and S-palmitoylation are protective. Subtoxic levels of ROS can sensitise the mPTP to low Ca^2+^ levels. Notably, this synergistic effect was observed in mitochondria lacking CypD, indicating that ROS-dependent mPTP sensitisation involves protein modifications beyond CypD [25]. Global C202S CypD knock-in mice, in which CypD-C202 was mutated to a serine to reduce oxidative modifications, exhibited desensitised mPTP opening and milder cardiac ischaemia/reperfusion injury [46]. In addition to post-translational modifications, CypD transcriptional regulation has garnered increasing attention. BMP/Smad signalling, a major driver of differentiation, was identified as a transcriptional repressor of the CypD gene *Ppif* [49]. This transcriptional regulatory effect inhibits mPTP opening and promotes osteogenic differentiation of bone marrow-derived mesenchymal stem cells (BMSCs). On the other hand, adipogenic C/EBPα functions as a transcriptional activator of *Ppif* to activate the mPTP in the adipogenic process of BMSCs [50]. Thus, it is evident that modulation of the mPTP open state through transcriptional regulation of the CypD gene *Ppif* can regulate the direction of stem cell differentiation.

It is important to note that CypD plays a multifaceted role in cellular signalling and metabolism beyond its involvement in mPTP formation. CypD binds peroxisome proliferator-activated receptor alpha (PPARα) and reduces PPARα activity to downregulate fatty acid β-oxidation in cisplatin acute kidney injury [51]. Under oxidative stress in cardiomyocytes, the PPARα–CypD interaction increases, but metformin can prevent this interaction via AMPK activation, which also reduces mPTP formation [52]. However, further studies are required to clarify a cause–effect relationship between PPARα–CypD interaction and mPTP opening. CypD interacts with Hsp60, a mitochondrial chaperone essential for the proper folding of mitochondrial proteins. Deletion of Hsp60 increased cell apoptosis and could be partially rescued by CsA treatment in yolk sac erythropoiesis [53]. Interestingly, Hsp60 knockdown under heat stress did not affect the expression of mitochondrial membrane proteins, including CypD, ANT, and PiC, but triggered BAX translocation to the mitochondria, altering outer membrane permeability [54]. Awareness of these diverse roles of CypD is essential for determining whether its relationship with the mPTP is correlative or causal.

In addition, proteins, including but not limited to those that interact with CypD (e.g., SIRT3 [31] and P53 [55,56]) or modulate endogenous regulators (e.g., creatine kinase brain-type CKB [57]), can indirectly regulate the mPTP and integrate its activity into cell physiology via signalling networks.

### 2.2. Consequences of mPTP Opening

mPT is important for mitochondria-related pathways and has been implicated in both the regulation of physiological cellular processes and the development of pathological conditions [24]. The reversible opening of the mPTP is regulated by the equilibrium between positive and negative modulators, which becomes out of balance under various cellular conditions, making the mPTP opening irreversible. Different statuses of mPTP opening can result in diverse consequences ranging from regulation of physiological events, such as Ca^2+^ homeostasis, mitochondrial membrane potential, and oxidative phosphorylation (OXPHOS), to induction of pathological events, such as mitochondrial dysfunction, ATP depletion, inflammation, mtDNA release, and cell death [49,58].

#### 2.2.1. The Physiological Roles of the mPTP

In recent years, insight into the role of the mPTP in physiological conditions has increased. Most research groups now believe that only the high-conductance and sustained mPTP opening is harmful, while the sub-conductance of the transient opening of the mPTP [59,60], called flickering, is necessary for mitochondrial calcium homeostasis [61,62], ROS signalling [63], mitochondrial membrane potential, cellular metabolism, and epigenetic regulation [49,58,64]. Impaired mPTP flickering is believed to be involved in the pathological mechanisms in several disease paradigms [64,65]. In addition, mPTP opening was demonstrated to affect the fate of stem cells via a metabolic and redox mechanism [36,66]. Current research indicates that spontaneous mPTP opening favours the maintenance of stem cell pluripotency, while mPTP closure may promote differentiation. During mouse cardiac development, mPTP closure increases mitochondrial maturation, decreases ROS accumulation, induces myocyte differentiation, and increases the cardiac function of neonatal mice [36,67]. Conversely, during the early phase of somatic cell reprogramming to induced pluripotent stem cells, short-term mPTP opening is thought to enhance reprogramming [58].

Mechanistically, this flickering leads to small-scale molecular exchanges within and outside the inner mitochondrial membrane, including Ca^2+^, ROS efflux from the mitochondria, and proton influx into the mitochondrial matrix [68,69]. mPTP flickering leads to localised transient depolarisation of the mitochondrial membrane, accompanied by transient mitochondrial Ca^2+^ release [61]. Inhibition of mPTP flickering by means of genetic deletion of *Ppif* (encoding CypD) leads to elevated resting Ca^2+^ levels in the matrix [64]. Mediated by mPTP flickering, the Ca^2+^ efflux limits the activation of the Ca^2+^-dependent TCA cycle [70], while the proton influx supports the aerobic glycolytic profile [71]. The mitochondrial OXPHOS function is impaired when the mPTP is open and is improved when this pore is kept closed by genetic deletion of CypD [72]. This suggests that physiological mPTP opening favours aerobic glycolysis over oxidative phosphorylation in cellular metabolism. Additionally, mPTP flickering is involved in mitophagy activation, since mPTP inhibition by means of pretreatment with CsA completely blocks mitochondrial Ca^2+^ oscillations and prevents Parkin recruitment to the outer mitochondrial membrane [73]. During the reprogramming process, mPTP flickering-mediated mitochondrial ROS efflux upregulates miR-101c expression, which induces PHF8-mediated H3K9me2/H3K27me3 demethylation of pluripotency genes. This provides us with a novel perspective for exploring the roles of metabolic reprogramming and ROS signalling in determining the fate of stem cells.

#### 2.2.2. Interaction Between mPTP Opening and Inflammation

On the one hand, mPTP opening provokes inflammation. In many bone-related diseases, excessively accumulated mtDNA in the cytoplasm acts as a damage-associated molecular pattern (DAMP) to trigger innate immunity and inflammation, activating cGMP AMP synthase (cGAS), a stimulator of interferon genes (STING) and inflammasome complexes, which enhances the production of pro-inflammatory cytokines. It has been demonstrated that the mechanism by which mtDNA fragments traverse the typically impermeable IMM to reach the cytoplasm is associated with the mPTP [74,75]. Opening of the mPTP occurs in response to NLRP3 agonists or TDP-43, allowing for mtDNA or oxidised mtDNA leakage into the cytoplasm [76,77]. Indeed, pharmacological inhibition of mPTP opening with CsA reduced the cytosolic pools of oxidised mtDNA by 60% ± 6% and mtDNA by 30–40% [76]. mPTP over-opening and pro-inflammatory gene overexpression induced by NLRP3 agonists or lipopolysaccharide (LPS) were significantly downregulated by CsA in bone marrow-derived macrophages (BMDMs) [76,78]. Such mPT may be initiated by mitochondrial calcium uniporter (MCU)-dependent mitochondrial calcium overload [76] and is not dependent on mitochondrial ROS [78].

On the other hand, mPTP opening is involved in inflammatory-induced cell dysfunction. Increased mPTP opening was found to occur during the RCD process activated by prototypical pro-inflammatory cytokines, such as tumour necrosis factor (TNF), interferon-γ (IFNγ), interleukin-1 (IL-1), and IL-6 [11,79]. Knocking down CypD by siRNA-*Ppif* interference or the addition of CsA rescued the mitochondrial function and osteogenic function of osteoblasts under TNF-α treatment, indicating the protective effects of blocking the CypD–mPTP axis against inflammatory mitochondrial dysfunction and bone damage [30].

#### 2.2.3. mPTP-Induced RCD

Triggered by extreme perturbations of intracellular homeostasis, such as severe oxidative stress and Ca^2+^ overload, the high-conductance current of mPT causes complete and persistent mitochondrial depolarisation, resulting in the activation of mitophagy or even cell death. mPT actively participates in RCD with necrotic or apoptotic features through the release of pro-apoptotic cofactors into the cytoplasm or the severe impairment of cell bioenergetics [24]. The widespread redistribution of ions eventually causes the influx of water and mitochondrial swelling, which activates the outer mitochondrial membrane (OMM) pore-forming proteins BAX and BAK, inducing mitochondrial outer membrane permeabilisation (MOMP) and allowing for the release of pro-apoptotic cofactors into the cytoplasm [24]. When ATP is limited, the cellular outcome favours necrosis over apoptosis. Apart from necrosis and apoptosis, detrimental mPT is involved in multiple other routes of RCD, including necroptosis [26,80], pyroptosis [81,82,83,84], ferroptosis [85,86], and autophagic cell death [87].

## 3. The mPTP and the Function of Bone-Related Cells

**Figure 2:**
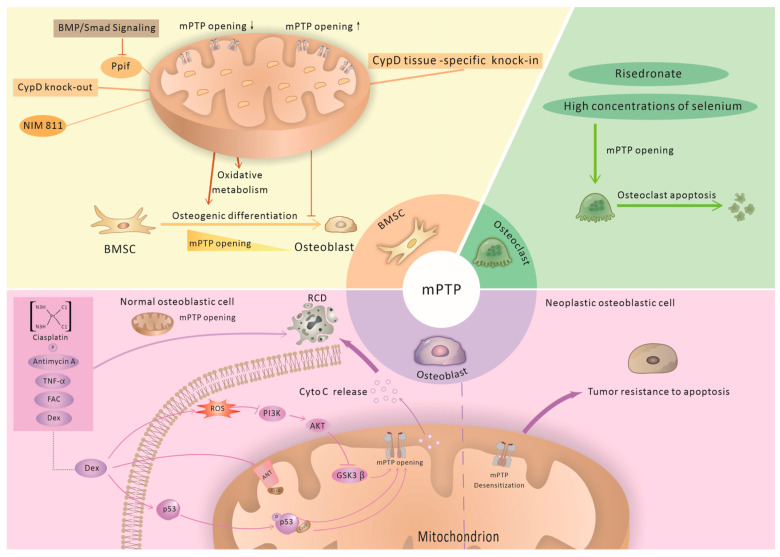
Diverse roles of mPTP opening in bone-related cells. The mitochondrial permeability transition pore (mPTP) is crucial in various functions within bone-related cells. In bone marrow-derived mesenchymal stem cells (BMSCs), cyclophilin D (CypD) downregulation and subsequent mPTP activity decline are required for osteogenic differentiation. In osteoblasts, mPTP over-opening is involved in several drug-induced regulated cell deaths (RCDs) in an ROS-dependent or ROS-independent manner. The mechanism by which osteosarcoma cells are resistant to apoptosis is, at least in part, due to desensitisation to the mPTP. Several studies have indicated that the mPTP is also involved in osteoclast apoptosis. ↑(black upward arrow) and ↓(black downward arrow) represents upregulation and downregulation of mPTP opening, respectively.

Investigations have suggested a link between the mPTP and the function of the osteogenic lineage. It is widely reported that cell energy metabolism shifts towards elevated mitochondrial OXPHOS during osteogenic differentiation [88,89,90,91]. Therefore, osteogenically differentiating BMSCs and their progeny, osteoblasts and osteocytes, are expected to be especially sensitive to mitochondrial dysfunction. It is reported that osteogenic differentiation is accompanied by the downregulation of *Ppif* mRNA expression in several osteogenic cell types, including BMSCs, ST2 cells, and MC3T3-E1 cells of mice [49]. In addition, mPTP opening actively participated in the process of cell death in bone-related cells (Figure 2).

### 3.1. The mPTP and BMSCs

Previous studies have revealed the important role of mPTP downregulation during the cell differentiation of various stem cells, including pluripotent stem cells [92] and neural [93], cardiac [36,67], and haematopoietic [94] cells. Recent studies have extended the important role of mPTP downregulation to the osteogenic lineage.

It has been demonstrated that CypD expression is downregulated and mPTP activity is decreased during osteogenic differentiation. BMP/Smad signalling, a major driver of differentiation in various lineages including the osteogenic lineage, transcriptionally represses *Ppif* promoter activity in osteogenic cells and consequently downregulates CypD expression during osteogenic differentiation [49]. BMSCs subjected to a calcein–cobalt assay showed decreased mPTP activity during osteogenic differentiation [49]. The BMSCs of CypD knock-out (KO) mice, a loss-of-function (LOF) model of the mPTP, showed higher BMSC OXPHOS function and osteogenic potential [95]. Specifically, when compared to control littermates, the BMSCs isolated from the CypD KO mice showed significantly higher mitochondrial membrane potential via CMXRos staining and a significantly higher oxygen consumption rate (OCR) in the Seahorse analysis [72]. Moreover, CypD KO BMSCs subjected to RNAseq transcriptome analysis and reactome pathway analysis showed significant upregulation of pro-osteogenic gene signatures, including genes involved in collagen formation, organisation, and maturation. Consistent with the pro-osteogenic transcriptomic signature, CypD KO BMSCs showed pronounced pro-osteogenic properties in the osteoinduction medium and in the BMP2-mediated ectopic bone (ossicle) formation assay in mice. Similar pro-osteogenic effects were exerted in BMSCs upon pharmacological inhibition of CypD with NIM811 [96]. Conversely, Sautchuk et al. recently designed a tissue-specific knock-in mouse model of CypD gain-of-function (GOF) that showed impaired BMSC osteogenic differentiation and mitochondrial function in osteogenic media, further establishing the necessity of CypD downregulation for OB differentiation [49].

### 3.2. The mPTP and Osteoblasts

Osteoblasts are the primary functional cells of bone formation and are responsible for the synthesis, secretion, and mineralisation of the bone matrix. Chronic or excessive use of glucocorticoids (GCs), such as dexamethasone (Dex), is a significant cause of secondary osteoporosis [97]. This is due, in part, to the death and apoptosis of osteoblasts induced by glucocorticoids [98]. Studies on the mechanism of Dex-induced osteoblast cell injury have revealed the critical role of mPTP opening. Dex considerably suppressed cell proliferation and stimulated apoptosis in rat primary osteoblasts and osteoblastic cell lines, including MC3T3-E1, human OB-6 osteoblastic cells, and human osteosarcoma MG63 cells. In Dex-treated MC3T3-E1 cells, CypD and ANT-1 binding increased, MMP decreased, and cytochrome C was released from mitochondria, suggesting an increase in mPTP opening. Notably, pretreatment with the mPTP inhibitor sanglifehrin A largely inhibited Dex-induced MC3T3-E1 cell death and the mPTP-opening-related events described above. Genetic and pharmacological inhibition of CypD suppressed Dex-induced MC3T3-E1 cell death, while CypD overexpression aggravated it.

At the molecular level, it was found that Dex induces P53 phosphorylation at serine 15 and translocation to mitochondria, where it forms a complex with CypD and triggers mPTP opening [56]. In primary murine osteoblasts and MC3T3-E1 cells, Dex suppressed the activation of PI3K/AKT signalling pathways by upregulating ROS levels [99]. Furthermore, pretreatment with SC79, a specific Akt activator, significantly attenuated Dex-induced mPTP opening, cytochrome C release, and apoptosis activation. Mechanistically, SC79 exerts this effect by directly inhibiting Dex-induced CypD–ANT-1 complexation [56,100] and potentially by attenuating Dex-induced oxidative stress via Akt-Nrf2 signalling [100]. Additionally, GSK-3β was shown to stimulate mPTP opening [101,102,103], and Dex dramatically upregulated GSK3β mRNA expression in osteoblasts and MC3T3-E1 cells [99]. However, whether and how GSK3β-induced mPTP opening is involved in Dex-induced osteoblast apoptosis remain uncertain. Although the underlying molecular mechanisms must be further investigated, the mPTP was implicated in the Dex-induced death of osteoblasts.

In addition to Dex, several other drugs have protective or damaging effects on osteoblasts by regulating the mPTP. Sciadopitysin, a type of biflavonoid, may prevent mPTP opening and osteoblast death triggered by an antimycin A-induced augment of ROS in MC3T3-E1 cells [104]. The anticarcinogen cisplatin is known to cause mitochondrial dysfunction, ROS-induced oxidative damage, and consequent mitochondria-mediated apoptosis in the same cell line. However, these effects can be mitigated by inhibiting mPTP opening [105].

One of the primary by-products of bone resorption, inorganic phosphate, may potentially elevate osteoblast apoptosis levels at the bone resorption location via mPT induction [106,107]. Genetic ablation or pharmacological inhibition of CypD through CsA rescued the mitochondrial and osteogenic function of osteoblasts when exposed to TNF-α treatment [30]. Iron overload induced by ferric ammonium citrate (FAC) treatment led to increased opening of the mPTP in osteoblastic cells, indicating that mPTP opening may be associated with osteoblastic necroptosis induced by iron overload [108].

Desensitisation of the mPTP may be a mechanistic basis for increased resistance to apoptosis of neoplastic cells. Osteosarcoma SAOS-2 cells displayed marked resistance to death caused by apoptotic stimuli, like arachidonic acid and the BH3 mimetic EM20-25, and this resistance was due to mitochondrial ERK activation that desensitises the PTP through a signalling axis involving GSK-3 and CypD [109]. By binding with ANT3 to inhibit the opening of the mPTP, EF-hand domain-containing protein 1 promotes the cell proliferation and drug resistance of the osteosarcoma cell line 143B [110]. The mPTP opener atractyloside was reported to decrease mitochondrial Ca^2+^ (mtCa^2+^) and sensitise osteosarcoma cells to TNF-related apoptosis-inducing ligand (TRAIL) cytotoxicity [111]. This indicates that the mPTP can be a promising target for overcoming the chemoresistance of osteosarcoma cells.

### 3.3. The mPTP and Osteoclasts

Risedronate, a nitrogen-containing bisphosphonate, is proficient in hindering bone resorption via osteoclast apoptosis induction. In human macrophage cell line U937 cells, risedronate-induced apoptosis is dependent on the mPT, as CsA and trifluoperazine, the potent inhibitors, effectively suppress the process [112]. High concentrations of selenium have a cytotoxic impact on osteoclast-like cells differentiated from RAW 264.7 cells, which provides evidence that CsA can hinder selenite-induced DNA fragmentation and apoptosis [113]. As previously stated, SIRT3 can desensitise mPTP via CypD demethylation at K166. However, SIRT3-KO mice showed an increased protein content of CypD and an elevated quantity of TRAP-positive osteoclasts in alveolar bone in comparison to controls [48]. This increased osteoclast activity could be attributed to the existence of osteoblasts and their considerable decrease in OPG production. These studies indicate that the potential side effects of drugs and the intercellular interaction must be considered when devising strategies to enhance bone phenotypes through inhibition of mPTP opening.

## 4. The Role of the mPTP in Bone-Related Pathological Conditions

Recent studies have gradually revealed the close relationship between mitochondrial dysfunction and bone-related diseases, but research on the role of the mPTP in this process is limited. Knock-out of CypD and pharmacological inhibition of CypD have demonstrated that mPTP desensitisation can lead to the amelioration of bone-related pathological conditions in animal models. Table 1 provides a summary of the evidence supporting the involvement of the mPTP in various bone-related pathological conditions.

### 4.1. Inhibition of the mPTP Improves Bone Healing

In a study using a mouse bone fracture model, the bone formation and biomechanical properties of repaired bones were significantly increased in mice with a pharmacological inhibition (using NIM811) or genetic deletion of CypD when compared to controls. Notably, these differences were evident in young male but not female mice, whereas older (13 months) female mice also showed increased bone formation properties when treated with NIM811, indicating that the inefficiency of the protective effect of mPTP inhibition in young female mice might be partly attributed to the inhibitory effect of oestrogen on the mPTP [115]. However, specific deletion of CypD in the mesenchymal lineage (Prx1-Cre driven) is not sufficient to improve bone fracture healing, which supports the use of global CypD KO and systemic pharmacological inhibitors [96]. CypD KO mice were used as a genetic model of high oxidative metabolism to investigate the effect of BMSC oxidative metabolism on graft osseointegration during spinal fusion. Compared to controls, CypD KO mice had greater mineralisation of the spinal fusion bridge, suggesting that inhibition of mPT may improve spinal fusion outcomes by promoting oxidative metabolism in osteogenic cells [72].

### 4.2. The Role of the mPTP in Osteoporosis

Osteoporosis, a systemic skeletal disease characterised by low-trauma fractures, is related to multiple mechanisms, including age-related bone loss, oestrogen deficiency, an increase in ROS, chronic inflammation, and weight loss [116,117,118].

Ageing is a complex phenomenon accompanied by mitochondrial deterioration. In particular, the opening threshold of the mPTP in old mice is lower than that in young mice [11]. mPTP involvement has been well established in the degenerative evolution of the brain [119], heart [64], and muscle [120]. Several animal studies have confirmed the involvement of CypD in age-related bone loss. Bones from aged mice present a higher protein abundance of CypD when compared to young mice. Meanwhile, mitochondria are impaired in ageing bone, manifested by a glycolytic shift, nucleotide imbalance, and decreased NAD/NADH ratio [48,95]. In terms of bone phenotype, there was a significant decrease in bone mass and strength at 13 months as compared to 3 months. Mitochondrial swelling (a marker of increased mPTP activity) was observed in the osteocytes of bones from 13-month-old mice, and CypD deletion protected against this mitochondrial dysfunction. In contrast, CypD KO mice showed enhanced resistance to age-related bone loss (at 13 and 18 months) and to metabolic changes observed in aged bone when compared to wild-type control littermate mice [95]. Apart from CypD global deletion [95], osteoblast-specific CypD deletion [49] in mice also protects against age-related bone loss in aged mice. Re-expression of CypD in OB-specific CypD-deficient mice by tibial intramedullary viral infection carrying a ca*Ppif* transgene revived the bone mechanical properties observed during ageing. Furthermore, CypD re-expression and gain-of-function (GOF) also led to decreased osteocalcin immunofluorescence (IF) and impaired bone phenotype in ageing [49]. Further study has shown that osteoblast-specific CypD GOF led to decreased OXPHOS levels, increased oxidative stress, and metabolic adaptations favouring decreased bone organic matrix content [114]. As indicated by previously published transcriptomic data and bone samples from 3- and 18-month-old mice, downregulation of BMP signalling was accompanied by significant upregulation of the CypD gene *Ppif* in aged mouse bones. In addition, the expression of SIRT3, an inhibitor of the mPTP via deacetylation of CypD, was decreased in the alveolar bone of the aged mice. The aged SIRT3-KO mice showed the highest degree of alveolar bone resorption and the largest number of active osteoclasts, accompanied by the highest protein abundance of CypD, compared to the young wild-type (WT), the aged WT, and the young SIRT3-OK mice [48]. Taken together, these results established the protective role of CypD/mPTP against age-related osteoporosis.

In addition, the mPTP was reported to be involved in certain drug-related osteoporosis mainly via the trigger of osteoblast cell death. Overuse of GCs (i.e., dexamethasone (Dex)) is the leading cause of secondary osteoporosis. CypD expression level significantly increased under Dex treatment. Pharmaceutical inhibition of CypD reduced oxidative stress accumulation in Dex-treated gingival tissue, suggesting that mPTP opening might be involved in Dex-induced oxidative stress [97]. Oxidative stress, in turn, is the potential upstream signal of Dex-induced mPTP opening, as evidenced by unpublished data showing that ROS scavengers largely inhibited Dex-induced mPTP opening and the following osteoblast cell death [100].

In conclusion, pathogenic factors associated with osteoporosis, such as ageing and oxidative stress, at least partly converge on mitochondria and impair their function via opening of the mPTP.

### 4.3. The mPTP and Bone Defects in Diabetes

The main mechanism that contributes to bone loss and delayed bone healing in diabetes is the production and accumulation of advanced glycation end-products (AGEs) and their major precursor, methylglyoxal (MG) [121,122,123]. A major mechanism by which AGE and MG negatively affect bone tissue is their induction of apoptosis in several osteogenic cell types, including human mesenchymal stem cells [124] and osteoblastic cells [125,126,127]. The addition of CsA significantly attenuated the apoptosis of MC3T3-E1 cells compared to AGEs alone, accompanied by the upregulation of Bcl-2 expression and downregulation of BAX expression. Similarly, the addition of MitoQ, a mitochondria-targeted antioxidant, rescued AGE-induced apoptosis. Mechanistically, AGEs significantly increased the production of intracellular [128] and mitochondrial [127] ROS in MC3T3-E1 cells. Inhibition of the mPTP by CsA restored mitochondrial membrane potential, ATP production, morphological abnormality, and perturbations of mitochondrial fission/fusion proteins elicited by AGEs. Suh et al. demonstrated that methylglyoxal toxicity in MC3T3-E1 cells was caused by oxidative stress and mitochondrial dysfunction [125]. In addition, diabetic serum (DS) treatment for 24 h significantly increased the level of ROS, H_2_O_2_, and mPTP opening activity in human BMSCs, accompanied by decreased cell viability and diminished osteogenic differentiation and pro-angiogenic capacity, which was attenuated by peroxisome proliferator-activated receptor gamma coactivator 1alpha (PGC-1α) overexpression via antioxidative effects [129]. These results revealed that oxidative stress, mitochondrial dysfunction, and mPTP opening were involved in the process of apoptosis and dysfunction of osteogenic cells in diabetes. mPTP activation, ROS levels, and calcium mobilisation interacted and formed a vicious circle, inducing mitochondrial damage and enhancing NLRP3 inflammasome activation related to the receptor for the AGEs (RAGE) RAGE/NF-κB pathway in human nucleus pulposus cells. Inhibition of the mPTP by CsA attenuated the NLRP3-inflammasome activation induced by AGEs [130].

Taken together, the in vitro AGE, MG, and DS treatments demonstrated that the mPTP may be actively involved in diabetes-induced oxidative stress, mitochondrial dysfunction, NLRP3-inflammasome activation, and apoptosis in MSCs and osteoblasts. Pharmacological inhibition of the mPTP effectively rescued AGE- and DS-induced cytotoxicity and functional impairment of osteogenic differentiation in MSCs and osteoblasts. In a rat model with experimentally induced diabetes and periodontitis, elevating the local expression of SIRT3, which has an inhibitory effect on the mPTP [31], mitigated alveolar bone loss and oxidative stress [131]. Despite this evidence, the precise role of mPT in diabetes-related bone defects and its underlying mechanisms have not yet been directly validated in animal models.

### 4.4. The mPTP and Inflammatory Bone Loss

In the ligature-induced periodontitis (LIP) model, the periodontal tissue of the LIP group demonstrated elevated mPTP opening levels when compared to the control group, accompanied by an increase in mtCa^2+^, mtROS, and cROS. The regulation of mPTP over-opening in BMDMs was effectively managed by decreasing Ca^2+^ overload through the application of mitochondria-targeted nanoparticles. Through this modulating effect, the nanoparticles efficiently reduced alveolar bone loss in the LIP model by inhibiting inflammatory activation and osteoclast activity [78].

Osteoarthritis (OA), a prevalent joint disorder, is distinguished by cartilage degradation, osteophyte formation, and synovial inflammation. Hypoxia/reoxygenation (H/R) plays an important role in the pathogenesis of OA. Fibroblast-like synoviocytes (FLS) located in the inner synovial layer of the synovium, which is thought to be associated with cartilage degradation in osteoarthritis, are highly susceptible to H/R. In both inflammatory and non-inflammatory conditions, H/R promoted mPTP opening, the release of intracellular ROS, mitochondrial matrix swelling, outer membrane rupture, and a decrease in the cristae of FLS. The above mitochondrial disturbances were also induced by the administration of TNF-α stimulation alone. H/R induced the expression of catabolic factors and senescence-associated secretory phenotype (SASP)-related factors in FLS and activated the NF-κB and JNK signalling pathways [132,133]. The protective effect of inhibiting the excessive opening of the mPTP against hypoxia/reoxygenation and inflammatory damage has been validated, suggesting that the mPTP may likewise be a promising target for controlling OA progression.

## 5. Conclusions and Future Perspectives

The opening of the mPTP allows for the redistribution of ions and metabolites inside and outside the mitochondria, promotes the release of mtDNA from the mitochondria, promotes the release of apoptotic factors from the mitochondrial membrane space, etc. The opening of the mPTP is actively involved in a wide range of activities from the modulation of physiological functions to the promotion of pathological changes, such as cytosolic inflammatory damage, activation of tissue inflammation, and RCD.

The mPTP performs different functions in different osteoblast lineages. In BMSCs, downregulation of CypD and the subsequent decrease in mPTP opening are required for osteogenic differentiation. In an ROS-dependent or ROS-independent manner, the mPTP is involved in several drug-induced osteoblast and osteoclast deaths. The mechanism by which osteosarcoma cells are resistant to apoptosis is, at least in part, due to insensitivity to the mPTP. The effects of modulating the mPTP on different cell populations and direct interactions between cell populations should be considered when investigating the role of the mPTP in bone homeostasis.

Genetic and pharmacological inhibition of the mPTP enhances bone repair following damage in a mouse fracture and spinal fusion model. The opening of the mPTP contributes to the progress of drug-associated, age-associated osteoporosis and diabetic metabolite-induced osteoblast dysfunction. In periodontitis and osteoarthritis models, mPTP opening activates inflammation and exacerbates bone loss through oxidative stress. Within the confines of the current research, mPTP opening is implicated in numerous bone pathological processes. Hampering the opening of the mPTP may serve as a promising approach to alleviate bone-related diseases and expedite bone mending.

Currently, the role of the mPTP in bone tissue is studied mostly through pharmacological inhibition with CsA or the genetic deletion of CypD. It is important to note that both CypD and CsA have functions beyond their role in mPTP formation, such as lipid metabolism regulation and immunosuppressive function, which should be considered when assessing mPTP involvement. In recent years, new CypD inhibitors, such as NIM811 and ebselen, have been discovered and utilised. In conclusion, the structure of the mPTP remains under debate, and researchers should keep updated on emerging developments in mPTP structural research while actively adopting advanced assays and more targeted mPTP inhibitors. In addition, the “multi-pore model” of the mPTP and the redundancy of mPT machinery may allow for a more moderate inhibition of the mPTP, sufficient to prevent its excessive opening under pathological conditions without being strong enough to interfere with its essential physiological functions.

## Figures and Tables

**Table 1:** Evidence for the involvement of the mPTP in bone-related pathological conditions.

Disease Model	Species	Cell Type	Intervention Modalities for mPTP	Results	Mechanism	Ref.
Tibial bone fracture model	C57BL/6J mice	BMSCs	Inhibition of mPT via genetic deletion of CypD; treatment with CypD inhibitor, NIM811	Increased bone formation and biomechanical properties; In contrast to global CypD knock-out, mesenchymal lineage-specific (Prx1-Cre driven) CypD deletion did not result in improved fracture repair	mPT inhibition promotes pro-osteogenic and anti-inflammatory effects in BMSCs	[96]
Posterolateral intertransverse lumbar fusion mouse model	C57BL/6J mice		Inhibition of mPT via genetic deletion of CypD	Increased bone formation and biomechanical properties; In contrast to global CypD knock-out, mesenchymal lineage-specific (Prx1-Cre driven) CypD deletion did not result in improved fracture repair	mPT inhibition promotes pro-osteogenic and anti-inflammatory effects in BMSCs	[72]
Ageing mouse model at age 16 months	129SV mice	-	-	Aged mice showed increased alveolar bone resorption with upregulated CypD expression	-	[48]
			Activation of mPT via SIRT3 germ-line knock-out	Increased alveolar bone resorption with upregulated CypD expression	SIRT3 deacetylates CypD and thus inhibits mPT	
Ageing mouse model at ages 13 and 18 months	Male C57BL/6J mice	-	-	Aged mice showed declined bone mass and strength with higher CypD expression and impaired mitochondria	-	[95]
			Inhibition of mPT via genetic deletion of CypD	Enhanced resistance to age-related bone loss and metabolic changes	-	
Ageing mouse model at age 18 months	C57BL/6J mice	-	Inhibition of mPT via OB-specific CypD deletion	Did not affect cortical thickness; Decreased age-related loss of trabecular bone volume and improved torsional rigidity	-	[58]
Ageing mouse model CypD GOF at age 24 months		-	OB-specific CypD/mPTP GOF via inserting caPpif cDNA encoding CypD K166Q mutant into the Rosa26 locus	Reversed the beneficial effects of CypD deletion on bone phenotype in aged mice	CypD/mPTP GOF disrupts mitochondrial function and impairs OB differentiation	
Ageing mouse model CypD GOF at age 12 months	-	BMSCs	OB-specific CypD/mPTP GOF via inserting caPpif cDNA encoding CypD K166Q mutant into the Rosa26 locus.	Decreased morphological and biomechanical properties; Decreased unmineralised matrix and proline content; Bone metabolome consistent with mitochondrial dysfunction, oxidative stress, decreased bone matrix anabolism, and premature ageing	CypD/mPTP GOF decreases osteogenic differentiation, collagen biosynthesis, mitochondrial OXPHOS parameters, and mitochondrial network in vitro	[114]
Ligature-induced periodontitis (LIP) model	Male C57BL/6 mice	BMDMs	-	Elevated mPTP opening levels, increased mtCa^2+^, mtROS, and cROS	mPT inhibition disrupts inflammatory activation and osteoclast activity	[78]
			Inhibition of mPT via treatment with CypD inhibitor, CsA treatment with VDAC inhibitor, VBIT4 decreasing Ca^2+^ overload by mitochondria-targeted nanoparticles	Reduced alveolar bone loss		

## Data Availability

Not applicable.
